# Assessment of Rumen Mucosa, Lung, and Liver Lesions at Slaughter as Benchmarking Tool for the Improvement of Finishing Beef Cattle Health and Welfare

**DOI:** 10.3389/fvets.2020.622837

**Published:** 2021-01-15

**Authors:** Luisa Magrin, Marta Brscic, Isabella Lora, Paola Prevedello, Barbara Contiero, Giulio Cozzi, Flaviana Gottardo

**Affiliations:** Department of Animal Medicine, Production and Health, University of Padova, Padova, Italy

**Keywords:** beef cattle, intensive production system, rumen hyperkeratosis, star scar, pneumonia, liver disease, *post-mortem* inspection, benchmarking system

## Abstract

Abattoir *post-mortem* inspections offer a useful tool for animal disease surveillance. The present cross-sectional study aimed at assessing the prevalence of rumen mucosa, lung, and liver lesions in 153 randomly selected batches of finishing beef cattle through a *post-mortem* inspection at the abattoir. At least 15 animals per batch were inspected at slaughter by two veterinarians for a total of 2,161 animals (1,376 bulls; 785 heifers) coming from 80 Italian commercial farms. Rumens were inspected by recording as binary variables (presence/absence) signs of hyperkeratosis, ruminitis, ulcer, and star scars. Similarly, lungs were inspected for signs of pneumonia and livers for signs of lipidosis, abscesses, and/or adherence. Hyperkeratosis of the mucosa and signs of ruminitis were detected in 58 and 30% of the inspected rumens, respectively. Ruminal star scars were more prevalent in bulls than in heifers (18 vs. 11%; *P* < 0.05). Signs of severe pneumonia were observed in 10% of the lungs; abscess and/or adherence in 4% of the livers. Hyperkeratosis of rumen mucosa was correlated to signs of ruminitis, and signs of ruminitis were correlated to star scars. No correlations were found between hepatic lesions and any other rumen or lung disorders. The wide variability observed among batches for the prevalence of specific lesions suggested the development of a benchmarking system to provide feedback to the farm veterinarians, as these lesions can be reflective of a subclinical disease status not easy to be detected in the live animal. Quartiles of the batch prevalence of rumen, lung, and liver alterations (if ≥1%) were calculated as a benchmarking tool, and third quartile value was proposed as an alarm threshold for each lesion. The use of the benchmarking system could allow to allocate each inspected batch to a specific “health class.” Critical batches with a prevalence above the alarm threshold for a given lesion should be reported to veterinarians of the origin farms where actions should be taken in order to identify and lower the risk factors for that specific health issue. Knowledge of *post-mortem* inspection data along with the implementation of the proposed benchmarking system should help farm veterinarians to improve herd management from a health and welfare perspective.

## Introduction

*Post-mortem* assessments at slaughter in cattle (1, 2), pigs (3), and poultry (4) have been recently considered a useful tool for animal disease surveillance. Inspections at slaughter offer the advantages of monitoring a large number of animals from several batches on the same day and collecting data from different organs in a reasonable time. This type of evaluation appears particularly cost- and labor-effective for the assessment of several digestive disorders or multiorgan diseases that could remain partially undetected *in vivo*. As in the intensive beef production systems, the appearance of feeding and management disorders could have a poor clinical manifestation (5), and the outcomes of the inspection could indirectly reveal some critical aspects of the animals' management in their farm of origin. For instance, respiratory diseases that are the most commonly reported health and economic problem both in feedlots and in intensively finishing beef cattle units (6–8) are often undetected at subclinical level or underdiagnosed on farm (9). Recently, the evaluation of the signs of bovine respiratory disease at slaughter has been proposed as an effective tool to define their negative impact during the fattening cycle (8, 10).

With regard to digestive disorders, subacute rumen acidosis (SARA) is considered an important issue for both beef cattle health and farm economy (11, 12). The development of SARA has been associated with the provision of high amounts of dietary non-structural carbohydrates (13) and/or of an insufficient amount of structured fiber (14). Recent findings in dairy cattle suggested that SARA is associated with a compromised rumen barrier and hindgut epithelium function that might allow toxin translocation and bacteria migration into the bloodstream, promoting local and systemic inflammation (15, 16). However, SARA diagnosis on beef farms is still challenging, since it is not associated with specific clinical signs (17). Ruminocentesis and rumen fluid analysis are direct diagnostic tools for SARA (18), but they are invasive and labor- and time-consuming, thus limiting the number of cattle that can be diagnosed. As an alternative to the direct on-farm diagnosis, *post-mortem* evaluation at slaughter of gross pathological evidence of rumen mucosa damage and specific liver alterations could be a retrospective strategy for the monitoring of beef cattle health, without any invasive handling on live animals.

Based on a *post-mortem* data collection at the abattoir from a wide range of randomly selected beef cattle batches coming from several fattening units, the present study aimed at (1) assessing the prevalence of different rumen mucosa, lung, and liver lesions; (2) calculating the potential correlations among different alterations detected on the same organ and among alterations detected on different organs; (3) developing a benchmarking system based on the prevalence of the recorded damage to drive cattle health improvements by farm veterinarians.

## Materials and Methods

A cross-sectional study was set up to gather information about the prevalence of rumen mucosa, lung, and liver alterations in finishing beef cattle at the slaughterhouse through a *post-mortem* inspection. Data collection was carried out in three commercial cattle slaughterhouses located in Northern Italy from April 2016 until March 2017. *Post-mortem* evaluations were carried out during 30 observation days on batches of cattle that were regularly slaughtered according to the ordinary slaughterhouse planning. Each observation day lasted from 06:00 h until 13:00 h with a target of inspecting at least six batches per day. A batch was considered a group of finished beef cattle of the same breed [Charolaise (CH), Limousine (LIM), or crossbreds (CR)] and category (bulls or heifers) coming from the same farm and belonging to the same slaughter group (same loading, transportation, unloading, lairage time, and slaughtering process). All farms of origin were located in the Po Valley (Italy), and their distance from the slaughterhouses was <3 h. *A priori*, it was set to inspect the organs of at least the first 15 animals per batch for batches larger than 15 animals, and of all the animals in case of smaller batches. This approach was used to trace the organs (rumen, lungs, and liver) of the same animal by two trained veterinarians located in different areas of the slaughterhouse (dispatch and tripery). The set of measures used for the study are described below and further detailed in Table 1. They have been chosen for their quick applicability (about 1 min/organ) without interfering with the regular working schedule of the slaughter line.

**Table 1:** The scoring system used for rumen, lung, and liver evaluation at slaughter in beef cattle.

**Specific disorder**	**Scale**	**Description**	**References**	**Supplementary figure**
**Rumen**				
Hyperkeratosis	0	Absence of the alteration	Hinders and Owen (19)	Supplementary Figure 1
	1	Hardened rumen papillae due to a thickening of the keratin layer, recorded after visual and tactile inspection		
Signs of ruminitis	0	Absence of the alteration	Thomson (20), Thompson et al. (21), Rezac et al. (1, 2)	Supplementary Figure 2
	1	Absent, rarified, or immature papillae with numerous whitish or reddish nodules of 2–3 mm		
Ulcer	0	Absence of the alteration	Thomson (20), Thompson et al. (21), Rezac et al. (1, 2)	
	1	Loss of integrity of the rumen mucosa with a severe perforation and inflammatory reaction		
Star scar	0	Absence of the alteration	Thomson (20), Thompson et al. (21), Rezac et al. (1, 2)	Supplementary Figure 3
	1	Star-shaped scar of lamellar keratin		
*Paramphistomum*	0	Absence of parasites		
	1	Presence of parasites		
**Lung**				
Pneumonia score	0	Healthy lung	Schneider et al. (10), Leruste et al. (22)	Supplementary Figure 4
	1	Minimal pneumonia = one spot (1–5 cm in diameter) of gray-red discoloration		
	2	Moderate pneumonia = one larger (>5 cm in diameter) or several small spots of gray-red discoloration with a total surface of about one lobe		
	3	Severe pneumonia = gray-red discoloration area involving more than one lobe		
**Liver**				
Signs of lipidosis	0	Absence of the alteration	Rezac et al. (1), Attia (12)	
	1	Rounded and enlarged ventral margins of the liver potentially indicating lipidosis		
Abscess and/or adherence	0	Absence of both alterations	Rezac et al. (1), Attia (12)	
	1	Presence of superficial abscess on liver or of fibrin adherence or presence of both, visible at inspection without resection. Adherence or other alterations on the liver surface were not resected in order to avoid contamination of the organs in case of abscesses located underneath		

### Organ Inspection

Rumens were inspected in the tripery after their dissection from intestines, omasa, and abomasa and their opening and emptying by the slaughterhouse operators. One trained veterinarian assessed rumen mucosa directly at the slaughter line having a water pump available to rinse the organ. Macroscopic alterations such as hyperkeratosis (Supplementary Figure 1), signs of ruminitis (Supplementary Figure 2), ulcers, and star scars (Supplementary Figure 3) were registered as binary measures (present/absent) following the methods adopted by previous studies and detailed in Table 1. Whenever there was presence of rumen parasites (*Paramphistomum*), it was also recorded as binary (presence/absence).

Using a simplified version of the assessment method described by previous researchers (Table 1), a second trained veterinarian examined lungs and livers directly in the slaughter line in the dispatch area. The assessor was positioned between the operator who detached the pluck from the carcass and the official veterinary inspector in order to visually and tactually inspect the organs before the official veterinarian did any cut or seizure. The assessor evaluated both lungs and attributed a score to the signs of pneumonia according to the description reported in Table 1 and Supplementary Figure 4 recording the worst condition of both lungs.

At the liver level, signs of lipidosis and the presence of abscesses and/or adherences were recorded as binary according to the criteria adapted from previous studies and reported in Table 1.

### Statistical Methods

The statistical analyses were carried out using SAS (9.3; Institute Inc., Cary, NC) and XLSTAT (Addinsoft, New York, NY). For binary variables, score 0 was used for the absence and score 1 for the presence of alteration. Batch was the experimental unit for all the prevalence of rumen, lung, and liver alterations. Per batch, the prevalence of rumen, lung, and liver alterations was calculated as the ratio of the number of organs with a specific score over the total number of organs inspected and expressed as a percentage. Normal distribution of the batch prevalence of all alterations was tested using the Shapiro–Wilk test.

When the batch prevalence for a given lesion resulted ≥1%, it was tested for the association with breed and gender. In particular, the prevalence of normally distributed data regarding lesions such as rumen hyperkeratosis, signs of ruminitis, and *Paramphistomum*, and lungs with minimal (Score 1) and moderate pneumonia (Score 2) were analyzed using a mixed model that considered breed, gender, and their interaction as fixed effects, and farm as random effect, with the Bonferroni adjustment option. Mann–Whitney test was performed to analyze the effect of gender, and Kruskal–Wallis test was used to analyze the effects of breed and gender × breed interaction for non-normally distributed data such as the prevalence of rumens with star scar, lungs with severe pneumonia (Score 3), and livers with abscess and/or adherence.

To find out the possible redundancies between different parameters recorded at the abattoir, correlations among the prevalence of lesions detected on the same organ or on different organs were assessed at batch level (with a batch prevalence ≥1%) using Spearman's rank correlation (PROC CORR of SAS 9.3; Institute Inc., Cary, NC).

Finally, quartiles of the batch prevalence of rumen, lung, and liver alterations (with a batch prevalence ≥1%) were calculated as a benchmarking tool, according to what was proposed by Scollo et al. (3) in pigs. The third quartile (Q3) value was proposed as an alarm threshold for each lesion, and specific quartiles were calculated within breed or gender when there was a significant effect of these factors on a given lesion.

## Results

Results of this study regard 2,161 animals (1,376 bulls and 785 heifers) belonging to 153 batches (97 bulls and 56 heifers) that came from 80 different fattening units (from one to seven batches/farm). The observed batches had a mean size of 29.9 ± 21.3 (SD) animals, and the average proportion of inspected animals per batch over the total number of animals within each batch was 65.5 ± 28.8%. Inspected cattle belonged to the following breeds: CH, 88 batches; LIM, 28; and CR, 37.

Almost 58 and 30% of the total inspected rumens showed hyperkeratosis or signs of ruminitis, respectively, and the prevalence of these lesions did not vary among breeds or genders (Table 2). Ulcers were detected only in 0.4% of the inspected rumens. The prevalence of rumens with star scars in the whole inspected sample was 15%, and it was higher for bulls than heifers (Table 2).

**Table 2:** Effects of breed, gender, and their interaction on the prevalence of specific organ lesions recorded *post-mortem* at the slaughterhouse in 153 batches of finishing beef cattle coming from 80 Italian commercial farms.

**Specific disorder**	**Overall^**1**^**	**Breed (B)**			**Gender (G)**		**Significance**		
		**CH**	**LIM**	**Other**	**Bull**	**Heifer**	**B**	**G**	**B × G**
**Rumen mucosa**									
Hyperkeratosis^2^	57.5	53.1 ± 3.0	58.5 ± 5.2	61.6 ± 4.3	58.9 ± 3.1	56.6 ± 4.0	ns	ns	ns
Signs of ruminitis^2^	29.9	25.9 ± 2.6	29.4 ± 4.8	35.6 ± 4.1	32.8 ± 2.7	27.9 ± 3.7	ns	ns	ns
Ulcer	0.4								
Star scar^3^	14.9	15.0 (11.1–18.8)	12.0 (7.9–16.1)	18.4 (10.9–25.9)	17.5^a^ (13.7–21.3)	11.4^b^ (6.9–15.9)	ns	*	ns
**Lung**									
Minimal pneumonia^2^	19.8	17.6 ± 1.4	21.7 ± 2.6	22.5 ± 2.3	21.9 ± 1.5	19.4 ± 2.1	ns	ns	ns
Moderate pneumonia^2^	9.3	9.3 ± 1.1	6.4 ± 1.9	11.1 ± 1.6	8.7 ± 1.1	9.2 ± 1.4	ns	ns	ns
Severe pneumonia^3^	10.3	9.7 (6.9–12.5)	12.7 (7.5–17.9)	10.2 (6.3–14.1)	10.6 (7.7–13.5)	9.9 (7.2–12.6)	ns	ns	ns
**Liver**									
Signs of lipidosis	0.1								
Abscess and/or adherence^3^	3.8	4.1 (2.8–5.4)	4.4 (2.3–6.4)	2.8 (1.1–4.5)	4.3 (3.1–5.6)	3.0 (1.6–4.4)	ns	ns	ns

1 Only disorders having an overall prevalence >1% were processed to test the effects of breed, gender, and their interaction.

2 Normally distributed variables were analyzed using a mixed model to test the effects of breed, gender, and their interaction, and their data are expressed as Lsmeans ± SEM.

3 Non-normally distributed variables were analyzed using non-parametric procedures to test the effects of breed, gender, and their interaction, and their data are expressed as mean and 95% confidence intervals.

a, b Values within a row with different superscripts differ (*P < 0.05).

*ns, not significant*.

The overall prevalence of rumens with *Paramphistomum* was 37%, and it differed among breeds, being higher (*P* < 0.001) for CH (41.6 ± 2.8%) and LIM (39.4 ± 4.8%) compared to CR (21.3 ± 3.8%). It was similar for bulls and heifer (34.2 ± 2.9% vs. 34.0 ± 3.6%, respectively).

Regarding lung score distribution, the overall prevalence of lungs scored 1 (minimal pneumonia) reached almost 20% of the inspected animals. The overall prevalence of lungs scored 2 and 3 (moderate and severe pneumonia) was around 10% of the inspected animals. None of these prevalence rates differed for breed or gender effect (Table 2). Over the total inspected livers, those showing signs of lipidosis were very rare (<0.5%) and those showing abscess and/or adherence were almost 4%. No effects of breed or gender were found for the prevalence of liver alterations (Table 2).

Results of the Spearman rank correlations on lesion prevalence at the batch level are reported in Table 3. The prevalence of rumen mucosa with hyperkeratosis was significantly correlated (*P* < 0.001) to that of rumens with signs of ruminitis, and it was negatively correlated (*P* < 0.05) to the prevalence of lungs showing severe signs of pneumonia. A positive correlation (*P* < 0.001) was also found between the prevalence of rumens with signs of ruminitis and that of rumens with star scars (Table 3).

**Table 3:** Correlations among different rumen mucosa, lung, and liver lesions recorded *post-mortem* at the slaughterhouse in 153 batches of finishing beef cattle coming from 80 Italian commercial farms.

**Specific disorder**	**Rumen mucosa**			**Lung**			**Liver**
	**Hyperkeratosis**	**Signs of ruminitis**	**Star scar**	**Minimal pneumonia**	**Moderate pneumonia**	**Severe pneumonia**	**Abscess and/or adherence**
**Rumen mucosa**							
Hyperkeratosis		0.284	0.054	0.130	−0.091	−0.170	−0.006
		***	ns	ns	ns	*	ns
Signs of ruminitis			0.439	0.004	0.038	−0.076	0.047
			***	ns	ns	ns	ns
Star scar				0.096	−0.151	−0.068	−0.076
				ns	ns	ns	ns
**Lung**							
Minimal pneumonia					−0.185	0.130	−0.099
					*	ns	ns
Moderate pneumonia						0.130	−0.100
						ns	ns
Severe pneumonia							0.062
							ns

Different symbols indicate significant correlations (*P <0.05 and ***P < 0.001).

*ns, not significant*.

**Figure 1:**
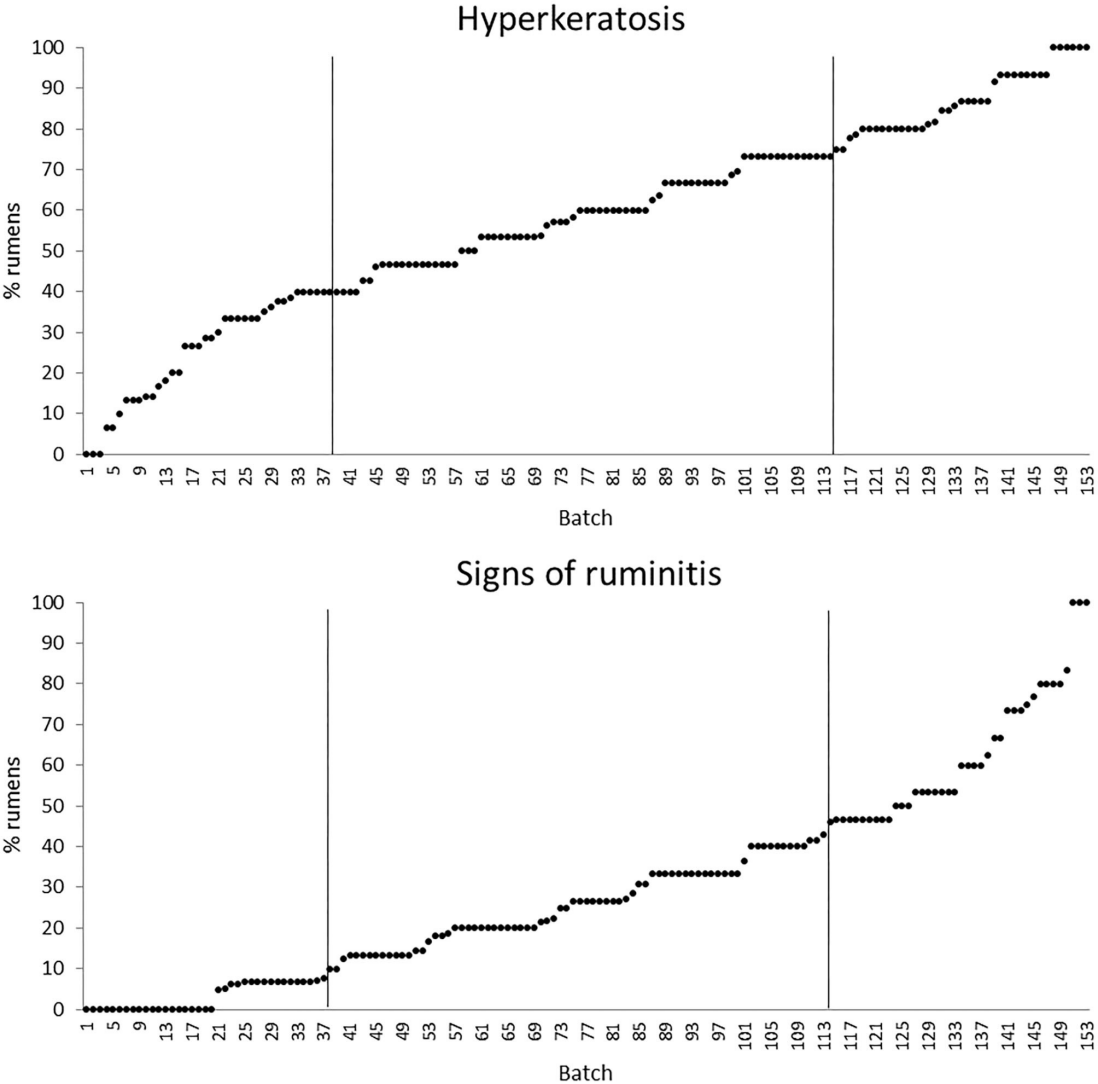
Distribution of the batch prevalence of rumen hyperkeratosis and signs of ruminitis of 153 batches of finishing beef cattle (represented by dots) coming from 80 Italian commercial farms. Solid vertical lines indicate thresholds at first and third quartiles.

**Figure 2:**
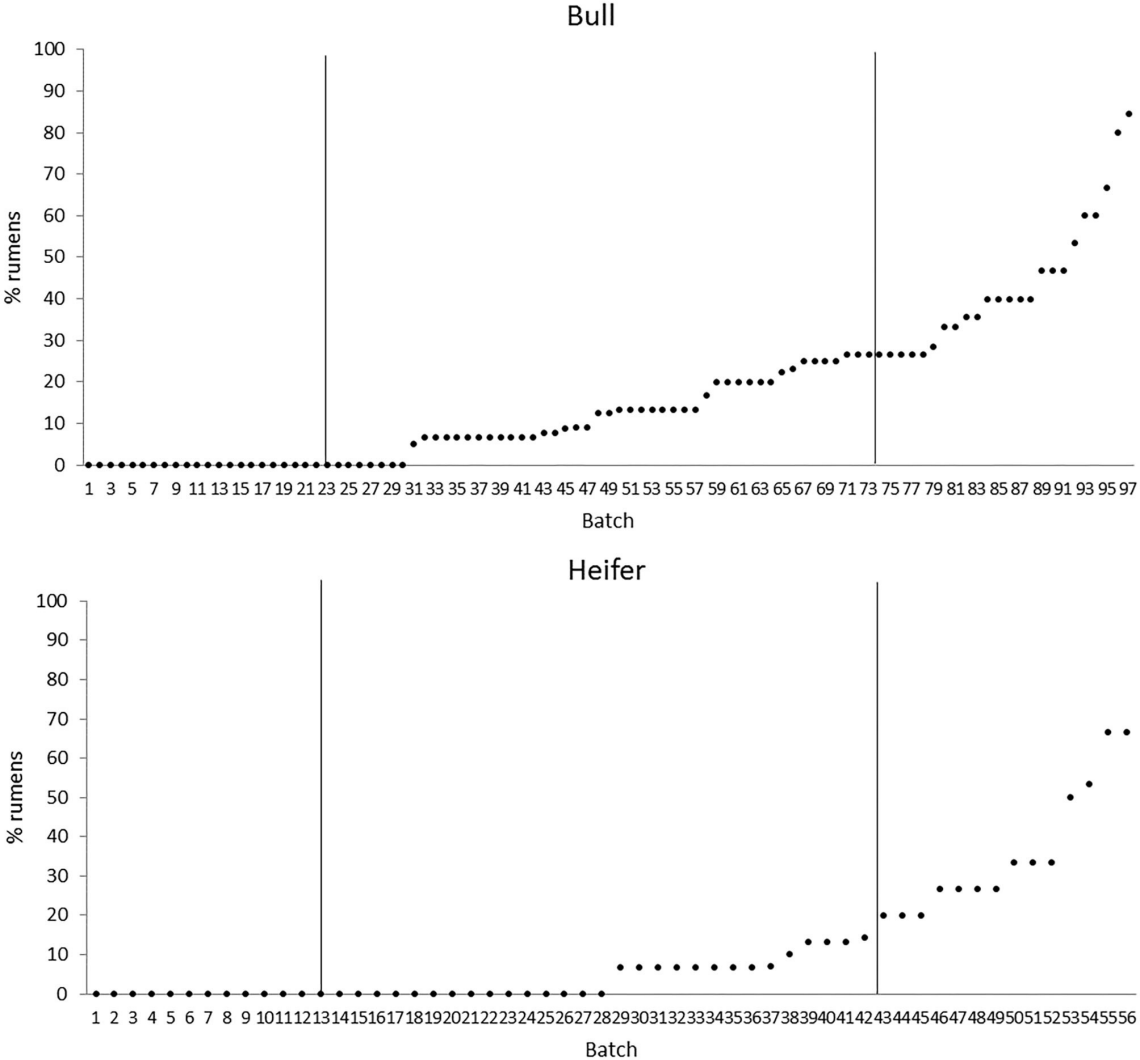
Distribution of the batch prevalence of rumen star scars of 153 batches of finishing beef cattle (represented by dots) coming from 80 Italian commercial farms divided by gender (bull, *n* = 97 batches; heifer, *n* = 56 batches). Solid vertical lines indicate thresholds at first and third quartiles.

**Figure 3:**
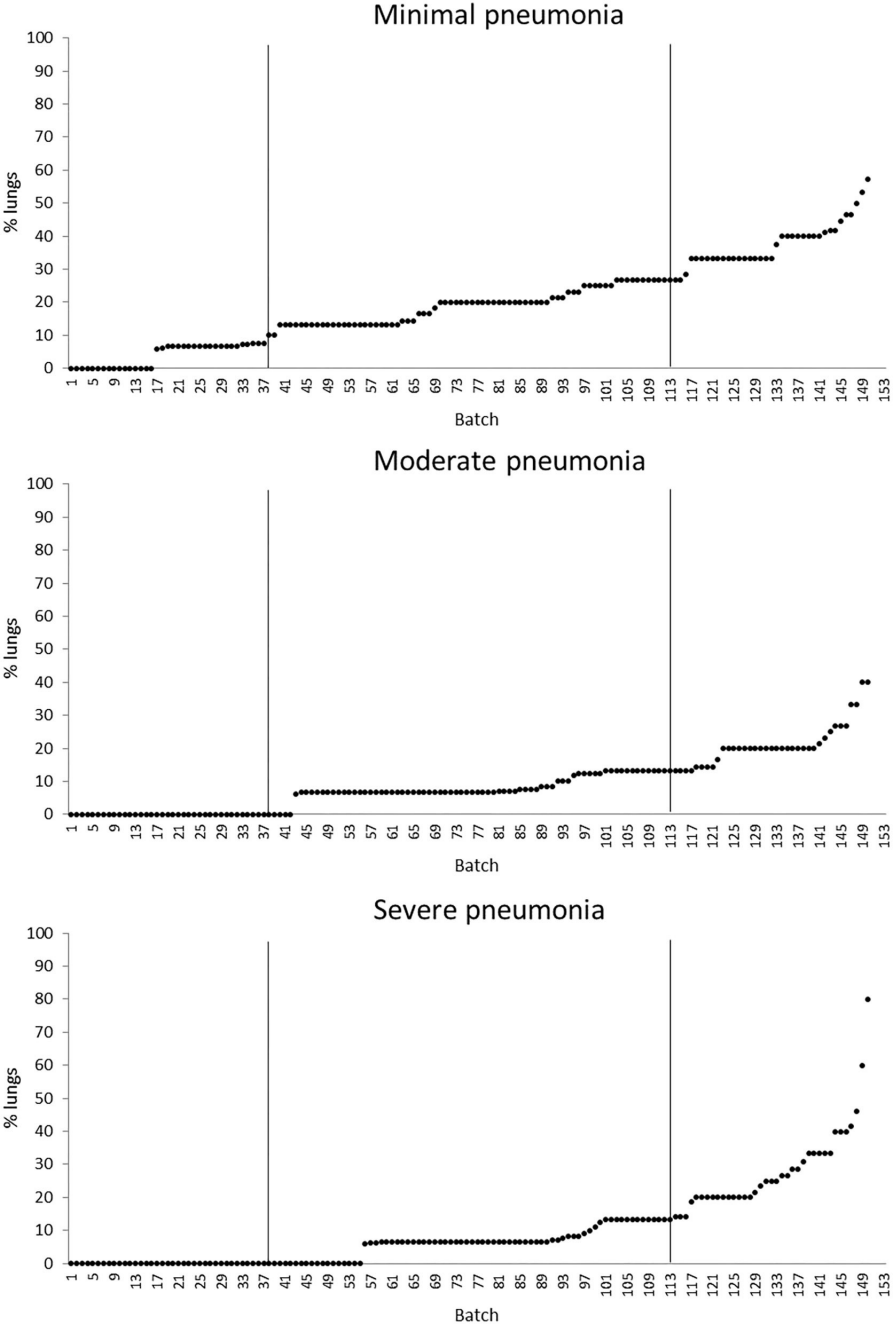
Distribution of the batch prevalence of lung minimal, moderate, and severe pneumonia of 153 batches of finishing beef cattle (represented by dots) coming from 80 Italian commercial farms. Solid vertical lines indicate thresholds at first and third quartiles.

**Figure 4:**
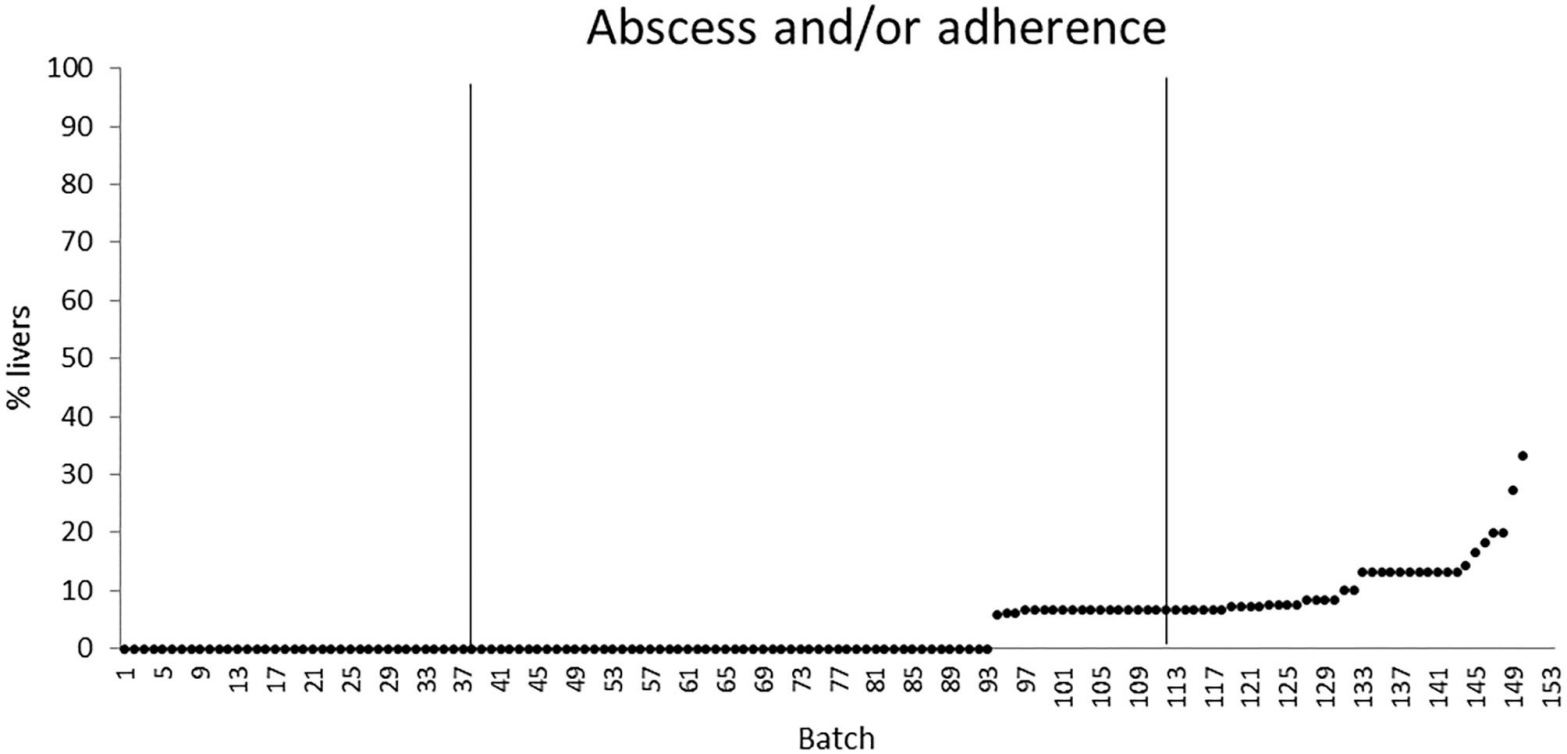
Distribution of the batch prevalence of liver abscess and/or adherence of 153 batches of finishing beef cattle (represented by dots) coming from 80 Italian commercial farms. Solid vertical lines indicate thresholds at first and third quartiles.

The quartiles of the prevalence of the specific lesions in the inspected batches are shown in Table 4. As the rumen hyperkeratosis was recorded with high frequency in most of the inspected batches, its median and Q3 values were very high, followed by the signs of ruminitis with a Q3 value of 47% (Figure 1). Batch prevalence of star scars differed between bulls and heifers, with the former having a higher alarm threshold value than the latter (27 vs. 16%, respectively; Figure 2). The prevalence of minimal signs of pneumonia at the batch level was higher than those of moderate and severe signs of pneumonia, leading to different alarm threshold values (Figure 3). The presence of abscesses or adherence in the liver was quite limited, with an alarm threshold value around 7% (Figure 4).

**Table 4:** Statistical description with quartiles of the batch average prevalence of specific organ lesions recorded *post-mortem* at the slaughterhouse in 153 batches of finishing beef cattle coming from 80 Italian commercial farms.

**Specific lesion (% of organs affected/batch)**	**Q1**	**Median**	**Q3**
**Rumen mucosa**			
Hyperkeratosis	40.0	60.0	75.0
Signs of ruminitis	10.0	26.7	46.7
Star scar			
Bull	0.0	12.5	26.7
Heifer	0.0	3.3	15.7
**Lung**			
Minimal pneumonia	10.0	20.0	26.7
Moderate pneumonia	0.0	6.7	13.3
Severe pneumonia	0.0	6.7	13.3
**Liver**			
Abscess and/or adherence	0.0	0.0	6.7

*Q1, first quartile; Q3, third quartile*.

## Discussion

The Italian beef cattle industry accounts for more than 1.5 million animals per year (5), nearly one third of the total beef cattle fattened in Europe. It is mainly located in the Po Valley, a fertile and homogeneous climatic area, and is based on the finishing of purebred or crossbred bulls and heifers imported from abroad at an age of 10–14 months and a live weight at the arrival of 300–400 kg (23). After their transfer to Italy, cattle are finished for about 7 months in specialized fattening units, housed in multiple pens with fully slatted or deep littered floor (24). Beef farms operate according to a rather standardized feeding program made of high-starch diets based on maize silage to promote maximum daily gain (25). Both housing and feeding plans have been recognized as potential risk factors for the health and welfare of the animals (1, 11), with increased costs for medical treatments (26).

The prevalence of rumen mucosa hyperkeratosis found in this study was considerably high and so widespread among batches that might be interpreted as an adaptive response of the animals to some common challenging conditions. Indeed, this alteration of the rumen wall is mainly associated with the provision of high-starch/low-fiber diets during the finishing period (27). At rumen level, the starch load might cause a temporary imbalance between production and absorption of fatty acids with a consequent drop in ruminal pH (14, 28). When a prolonged condition of low rumen pH (5.5–5.0) persists, bacteria might invade the rumen wall and eventually lead to ruminitis and severe damage to the rumen mucosa papillae (14, 29). The quite high prevalence of signs of ruminitis recorded in this study is consistent to this hypothesis. The relevant role of the dietary energy content in developing specific damage on the rumen wall has been proved by several studies on finishing feedlot cattle, where animals fed diets with more than 70% of concentrate in a finishing period of 3 months had an occurrence of rumen mucosa ulcers and/or ruminitis of 19.9–31.3% (1, 26).

Particularly alarming in the current study was the noticeable prevalence of star scars on the rumen mucosa that was similar to the values recorded in feedlot cattle (1, 26). Star scars represent the footprint of a previous pathological condition, as they are outcomes of the healing process from ruminitis or ulceration. Even their etiology arises from the ingestion of a high amount of grains or of high-starch/low-fiber rations that induce a relevant chemical insult to the ruminal mucosa with a reduction of its absorptive capacity (1, 2, 30). In the present study, the prevalence of star scars was higher for bulls than for heifers, and this result may be attributed to the different feeding plans adopted for these two cattle categories. Indeed, beef heifers are fed more roughages and fattened for a shorter period than beef bulls to prevent an excessive carcass fatness score (23).

In this study, the occasional finding of massive rumen infestation by parasites of the genus *Paramphistomum* was heavily influenced by cattle breeds likely reflecting their different origins. French CH and LIM are generally kept at pasture before the transfer to Italy and thus easily exposed to *Paramphistomum* infestation (31). Crossbred cattle are instead mainly of domestic origin, and they have a very limited or no access to pasture before finishing. *Paramphistomum* infestation usually has a subclinical course; however, the negative effect of a massive infestation on rumen health and absorptive efficiency should be carefully evaluated by further dedicated studies.

In literature, the reported prevalence of liver abscesses in feedlot cattle showed a great variability from 4.8% in McKeith et al. (32) to 13.9% in Garcia et al. (33), with the highest value of 32% reported by Nagaraja and Lechtenberg (34) for Canadian and North American cattle. The difference between these values and the prevalence recorded in the current study (about 4.0%) was presumably due to the diverse feeding regimen and environmental conditions (indoor vs. outdoor housing, climatic zone, etc.) that characterize these beef production systems. Some feedlot studies have proven the existence of an association between the occurrence of rumen pathologies and liver abscesses within the so-called ruminitis–liver abscess complex (1, 12). In our study, the low prevalence of liver abscesses and the lack of any association between them and the rumen lesions suggest that a possible SARA condition affecting the inspected batches did not induce a severe inflammatory response with the translocation of bacteria and endotoxins across the ruminal epithelium.

The overall prevalence of healthy lungs recorded in this study (on average 62.0%) was satisfactory if compared to the outcome (36%) of a similar study carried out on intensively finished beef cattle reared in the same area (8). Moreover, our percentage of healthy lungs was consistent with previous values recorded at the slaughter for feedlot cattle, which ranged from 60 to 72% (1, 10, 35). In these feedlot studies, the prevalence of clinical signs of 35 and 8.17% observed *in vivo* by Wittum et al. (35) and Schneider et al. (10), respectively, was significantly lower than that of lung lesions recorded *post-mortem*, indicating that direct *on-farm* diagnosis could neglect a large number of animals that have likely suffered from subclinical pneumonia (10). Therefore, as the evaluation of lungs at the abattoir is a reliable indicator of the real occurrence of respiratory diseases during the fattening period, data from *post-mortem* inspections should complement the *in vivo* health checks in order to detect specific risks that could impair the lungs' health on farm.

The improvement of animal health surveillance through the identification of simple reliable indicators is a priority of the EU animal health strategy (36). In this regard, *post-mortem* inspections at the abattoir are an important complement to the *intra vitam* health checks, as the recorded lesions can be reflective of subclinical disease status not easily detected in the live animal. In addition, a benchmarking system based on inspections data about the prevalence and severity of lesions at batch level could support farm veterinarians to prioritize their actions to improve the herd management from a health and welfare perspective.

Consistent with the approach proposed by Scollo et al. (3) in pigs, the calculation of the quartiles for the batch population according to the prevalence of each rumen, lung, and liver lesion allows to allocate each inspected batch in a specific “health class.” The rationale behind this benchmarking system should foresee a targeted intervention by the stockman and the veterinarian in those farms for which, at slaughter, batches of cattle showed a prevalence above the defined alarm threshold (Q3) for a given lesion. The implementation of the system through a wide collection of *post-mortem* organ inspection data in several abattoirs would set reliable alarm thresholds for each lesion for a specific country or production system. At this purpose, to limit a possible bias due to the interobserver variation, it would be advisable to organize a proper training to standardize the definitions of lesions and scores across the inspectors working in different abattoirs. A regular application of the benchmarking system should promote a virtuous cycle in which beef farms where a specific intervention is needed are taking actions to improve health and feeding management and move to a better quartile. In the medium to long run, the benchmarking system should support a general and continuous improvement of beef cattle health management, resulting in better performance and a lower use of pharmaceutical treatments.

This study provided an overview of the occurrence of some specific rumen, lung, and liver lesions that affect intensively finished beef cattle. Some of the recorded lesions, like rumen hyperkeratosis and signs of ruminitis, had a very high prevalence, suggesting the need for an intervention on the feeding management during the finishing period. Knowledge of *post-mortem* organ inspection data is of value, as several lesions can be reflective of subclinical disease status not easily detected in the live animal. The variability observed among the batch prevalence for specific signs of diseases suggested the development of a benchmarking system to help farm veterinarians to drive herd health improvement. A wide implementation of this system should promote a continuous improvement of beef cattle management from a health and welfare perspective.

## Data Availability Statement

The raw data supporting the conclusions of this article will be made available by the authors, without undue reservation.

## Ethics Statement

Ethical review and approval was not required for the animal study because the organs' assessments were performed post-mortem only.

## Author Contributions

FG and GC conceived and designed study. LM, MB, IL, and PP collected, compiled, and analyzed the data. BC performed statistical analyses. LM, IL, and GC drafted and edited the manuscript. All authors contributed to the article and approved the submitted version.

## Conflict of Interest

The authors declare that the research was conducted in the absence of any commercial or financial relationships that could be construed as a potential conflict of interest.
