# Supplementary figures and images for: Assessment of Rumen Mucosa, Lung, and Liver Lesions at Slaughter as Benchmarking Tool for the Improvement of Finishing Beef Cattle Health and Welfare
Source: Front Vet Sci. 2021 Jan 15;7:622837. doi: 10.3389/fvets.2020.622837 (PMC7843922; doi:10.3389/fvets.2020.622837)

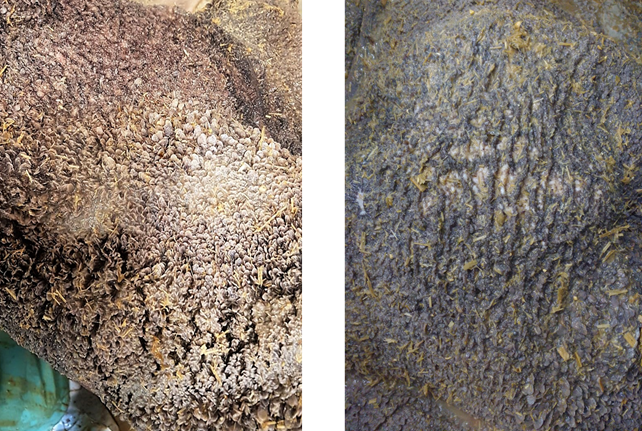

Supplement: Supplementary file 1 [file Image_1.TIF]

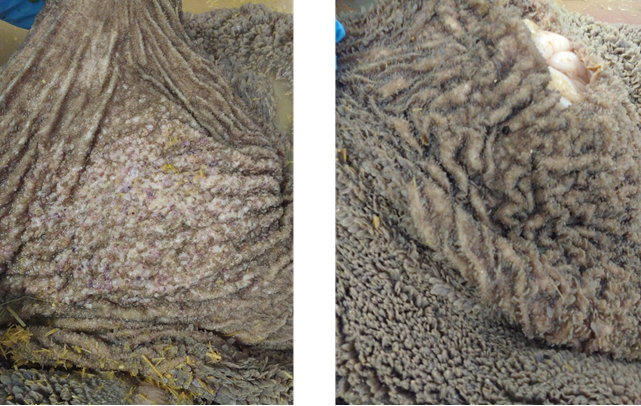

Supplement: Supplementary file 2 [file Image_2.TIF]

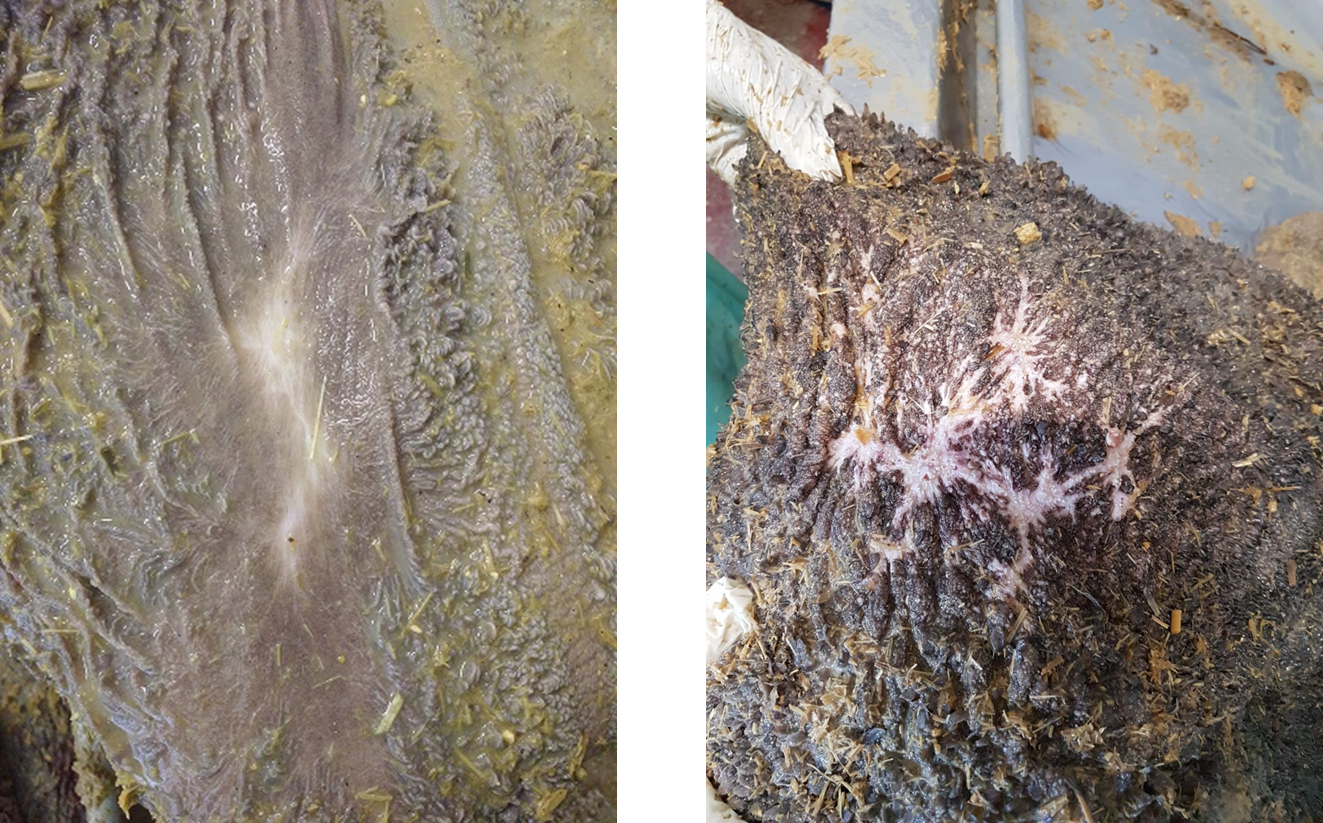

Supplement: Supplementary file 3 [file Image_3.TIF]

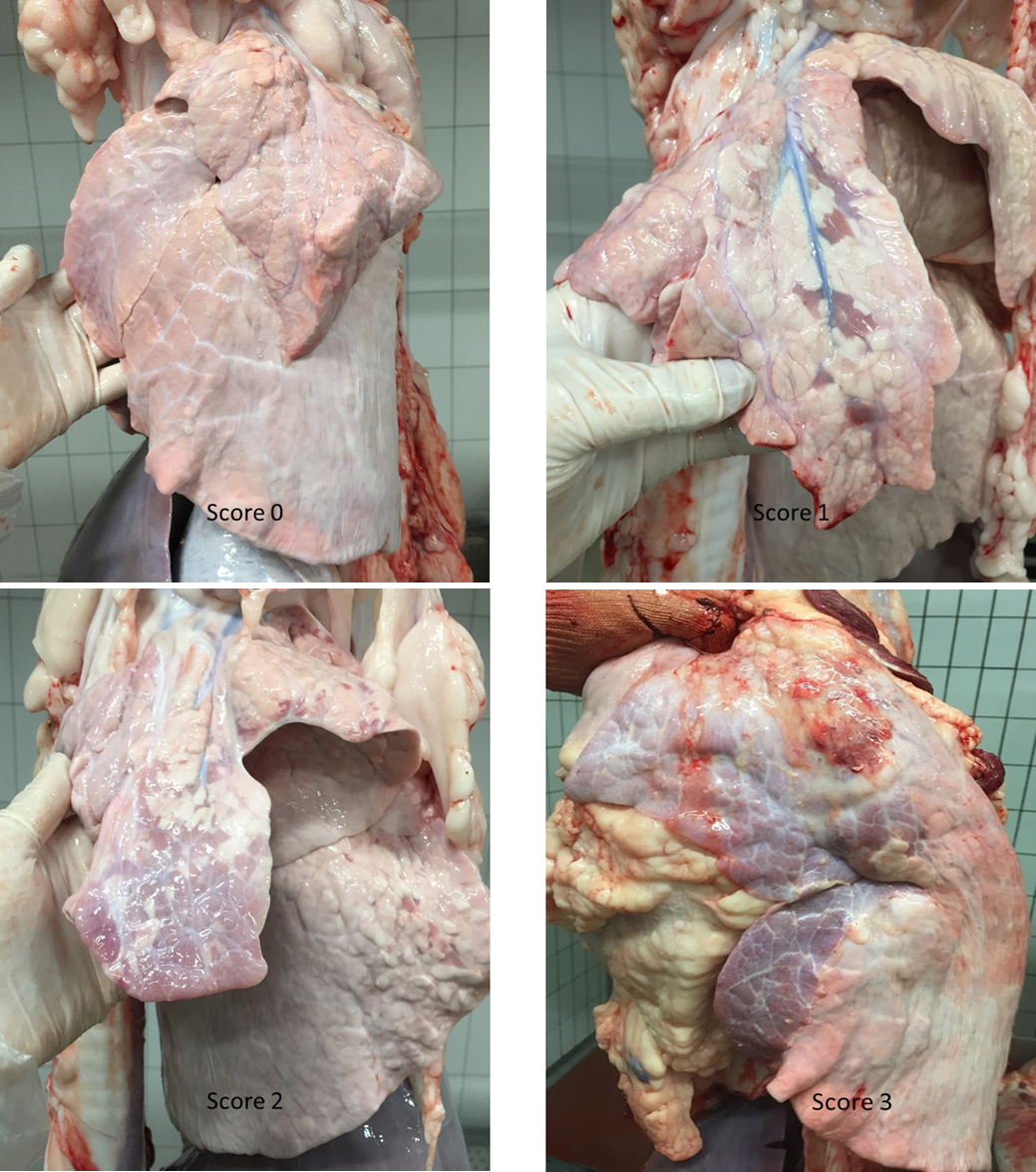

Supplement: Supplementary file 4 [file Image_4.TIF]
